# Dietary changes during weaning shape the gut microbiota of red pandas (*Ailurus fulgens*)

**DOI:** 10.1093/conphys/cox075

**Published:** 2018-01-06

**Authors:** Candace L Williams, Kimberly A Dill-McFarland, Darrell L Sparks, Andrew J Kouba, Scott T Willard, Garret Suen, Ashli E Brown

**Affiliations:** 1 Department of Biochemistry, Molecular Biology, Entomology and Plant Pathology, Mississippi State University, 32 Creelman Street, Mississippi State, MS 39762, USA; 2 Institute for Genomics, Biocomputing and Biotechnology, Mississippi State University, 2 Research Blvd, Box 9627, Mississippi State, MS 39762, USA; 3 Department of Bacteriology, University of Wisconsin-Madison, 1550 Linden Dr, Madison, WI 53706, USA; 4 Mississippi State Chemical Laboratory, 1145 Hand Lab, 310 Presidents Circle, Mississippi State, MS 39762, USA; 5 Department of Research and Conservation, Memphis Zoological Society, 2000 Prentiss Place, Memphis, TN 38112, USA

**Keywords:** Bacterial succession, gastrointestinal tract microbiota, herbivorous carnivore, microbiome, 16S rRNA parallel sequencing

## Abstract

Mammalian herbivores have developed numerous adaptations to utilize their plant-based diets including a modified gastrointestinal tract (GIT) and symbiosis with a GIT microbiota that plays a major role in digestion and the maintenance of host health. The red panda (*Ailurus fulgens*) is a herbivorous carnivore that lacks the specialized GIT common to other herbivores but still relies on microorganisms for survival on its almost entirely bamboo diet. The GIT microbiota is of further importance in young red pandas, as high cub mortality is problematic and has been attributed to failure to meet nutritional requirements. To gain insight into the establishment of the GIT microbiota of red pandas, we examined microbial communities in two individuals following dietary changes associated with weaning using next-generation 16S rRNA Illumina MiSeq paired-end sequencing of faecal samples. Across all four stages (pre-weaning, during weaning, post-weaning and adult), the GIT microbial community displayed low diversity and was dominated by bacteria in the phylum Firmicutes with lesser contributions from the Proteobacteria. A core community was found consistently across all weaning stages and included species within the taxa *Escherichia-Shigella, Streptococcus*, *Clostridium* and an unclassified Clostridiaceae. Analysis of the overall community composition and structure showed that although the GIT microbiota is established early in red pandas, dietary changes during weaning further shape the community and are correlated with the presence of new bacterial species. This work is the first analysis of the GIT microbiota for red panda cubs during weaning and provides a framework for understanding how diet and host microbiota impact the development of these threatened animals.

## Teaser

We examined red panda faecal microbial communities following dietary changes associated to weaning using 16S rRNA Illumina MiSeq paired-end sequencing, finding that the gut microbiota is established early, but dietary changes further shape the community with the presence of new bacterial species.

## Introduction

Microorganisms play a fundamental role in the survival of their animal hosts (McFall-Ngai *et al.*, 2013). Specifically, the gastrointestinal tract (GIT) microbiota maintains the host’s immune system through modulation of normal GIT function and pathogen exclusion as well as contributes to nutrient and energy acquisition (Hooper *et al.*, 2002; Flint *et al.*, 2012). Gut-associated microbes are particularly important in herbivores where they are wholly responsible for the breakdown of dietary plant matter into accessible nutrients (Bergman, 1990). Many herbivores have evolved a number of physiological adaptations, such as a rumen and slow rate of digestion, to promote and enhance this microbial fermentation. An unusual exception is the red panda (*Ailurus fulgens*), which is able to survive on entirely plant-derived materials without such adaptations. Specifically, red pandas are members of Carnivora but consume an exclusively herbivorous diet (Loeffler, 2011). Unlike typical herbivores, they have the GIT of a carnivore (Davis, 1964) with a simple stomach, no caecum (Davis, 1964; Roberts and Gittleman, 1984), and a shorter digesta transit time (the rate of ingested foods passing through the GIT) on the order of 10 h or less (Bleijenberg and Nijboer, 1989; Nijboer and Dierenfeld, 2011) compared to 48–75 h in traditional hindgut herbivores, such as equines (Cuddeford *et al.*, 1995), and 2–6 h for carnivores, such as canines (Boillat *et al.*, 2010).

Recent work on the GIT microbiota of both wild and captive adult red pandas found that the microbes present in the gut have some cellulolytic capabilities (Kong *et al.*, 2014). This indicates that, like other herbivores, gut microorganisms in red pandas may play an important role in nutrient provisioning. However, there is little information regarding the acquisition of these microbial communities in red panda cubs as they are weaned from a milk-based diet to bamboo. Given that there is no evidence that red pandas undergo extensive changes in GIT morphology (Davis, 1964), as seen in other herbivores, it is unclear how and when red pandas acquire the fibrolytic organisms necessary for survival as an adult.

This is particularly important for captive red pandas, as cubs born in North American institutions have high first year mortality (~50%), which has been attributed to numerous husbandry factors including a failure to meet nutritional requirements (Loeffler, 2011). This is extremely problematic, as red pandas are considered an endangered species with a decreasing population of 10 000 individuals (IUCN, 2015) and *ex situ* breeding programs that are not as successful as expected (Loeffler, 2011). Therefore, gaining an understanding of the developing GIT microbiota in young red pandas may lead to new insights for the husbandry of captive red pandas.

In this exploratory study, the faecal bacterial microbiotas of two captive red panda cubs were tracked through weaning using next-generation sequencing of the bacterial 16S rRNA gene in faecal material. Bacterial communities at different weaning stages (pre-weaning, during weaning, post-weaning and adult) were compared to determine succession into an adult microbiota. We also correlated these data to diet to determine its effect on the development of the red panda GIT microbiota during weaning. These data provide the first insights into the young red pandas GIT microbiota and informs on their peculiar biology as herbivorous carnivores.

## Materials and methods

### Study animals

All animals used in this study were housed at the Memphis Zoological Society, Memphis, TN, USA. Red panda faecal samples from the Memphis Zoo were collected under a signed biomaterials request form, and no IACUC was needed as the project was viewed as non-invasive by the institution. This study consisted of two hand-reared red pandas, a young breeding pair including: a young male, ‘Justin’ (Studbook number: 1219, Date of birth (DOB) July 1, 2012) and a young female, ‘Lucille’ (Studbook number: 1215, DOB 21 June 2012). Both remained healthy throughout the study duration and were housed at the Memphis Zoological Society according to the standard husbandry practices (AZA Small Carnivore TAG, 2012) with access to indoor and outdoor enclosures. Animals were co-housed and therefore, not all samples could be attributed to a specific individual.

### Sample collection

Fresh faecal samples were collected from red pandas following defecation at different stages of weaning. All samples were flash-frozen in liquid N_2_, transported on dry ice and stored at −80°C prior to processing. Sample collection times occurred in coordination with changes in diet composition at pre-weaning (Stage 1, *n* = 3), during the weaning transition (Stage 2, *n* = 3), post-weaning (Stage 3, *n* = 3) and adult (Stage 4, *n* = 6) (Table 1).

**Table 1:** Sampling period diet composition

Weaning stage	Date	Age (days)		Faecal samples (*n*)	Diet
		‘Lucille’	‘Justin’		
Stage 1	8/08/2012	38	48	3	Milk replacer (Esibilac)
Stage 2	10/29/2012	120	130	3	Milk replacer, leaf eater diet (Mazuri), bamboo introduced
Stage 3	2/12/2013	226	236	3	Bamboo, leaf eater diet
Stage 4	10/16/2013	472	482	6	Bamboo, leaf eater diet

### DNA extraction

Total genomic DNA from individual faecal samples was extracted via mechanical disruption and hot/cold phenol extraction following Stevenson and Weimer’s protocol (2007) with the exception that 25:24:1 phenol:chloroform:isoamyl alcohol was used in place of phenol:chloroform at all steps. DNA was quantified using a Qubit Fluorometer (Invitrogen, Carlsbad, CA, USA) and stored at −20°C following extraction.

### Library preparation and sequencing

Library preparation was carried out following manufacturer’s recommendations (Illumina, 2013) with some modifications. In brief, an amplicon PCR targeted the V3-V4 region of the 16S rRNA gene using a forward (V3-4F, TCGTCGGCAGCGTCAGATGT GTATAAGAGACAGCCTACGGGNGGCWGCAG) and reverse (V3-4R, GTCTCGTG GGCTCGGAGATGTGTATAAGAGACAGGC TACHVGGGTATCTAATCC) primers (Klindworth *et al.*, 2013) in a 25 μl reaction with 1× KAPA HiFi Hot Start Ready Mix (Kapa Biosystems, Wilmington, MA, USA), 0.2 mM of each primer and 1–10 ng DNA. Amplification conditions were as follows: 95°C for 3 min, 25 cycles of 95°C for 30 s, 55°C for 30 s, 72°C for 30 s and a final elongation of 72°C for 5 min. PCR products were purified via gel extraction (Zymo Gel DNA Recovery Kit; Zymo, Irvine, CA) from a 1.0% low melt agarose gel (National Diagnostics, Atlanta, GA). Purified products underwent a second 25 μl-PCR reaction to add unique indices (1× KAPA HiFi Hot Start Ready Mix, 0.2 mM indices and 5 μl of purified product) with the same reaction conditions as amplicon PCR with the exception of a reduction in the number of cycles to 8. The final indexed PCR product underwent gel extraction (Zymo Gel DNA Recovery Kit; Zymo, Irvine, CA), and the resulting purified product concentration was determined by a Qubit Fluorometer (Invitrogen Carlsbad, CA, USA). Samples were combined to yield an equimolar 4 nM pool. Following manufacturer’s protocol, sequencing was conducted on an Illumina MiSeq using reagent kit V3 (2 × 300 bp cycles), as described previously (Williams *et al.*, 2016). All sequences were deposited into the National Center for Biotechnological Information’s Short Read Archive under Accession Number SRP077938.

### Data analysis

Sequences were processed as described previously (Williams *et al.*, 2016) using the program mothur v.1.36.1 following the MiSeq SOP (Kozich *et al.*, 2013). In brief, contigs were formed from 16S rRNA reads, and poor quality sequences were removed. Sequences were trimmed and filtered based on quality (maxambig = 0, minlength = 250, maxlength = 600). Unique sequences were aligned against the SILVA 16S rRNA gene alignment database (Pruesse *et al.*, 2007) and classified with a bootstrap value cutoff of 80, and operational taxonomic units (OTUs) found with <2 sequences in the total dataset were removed. Chimeras (chimera.uchime) and sequences identified as members of Eukaryota, Archaea and Cyanobacteria lineages were also removed, and remaining sequences were clustered at 97% identity threshold.

### Statistical analysis

Sequence coverage was assessed in mothur by rarefaction curves and Good’s coverage (Good, 1953). Samples were then iteratively subsampled 10 times to 5511 sequences per sample (according to the size of our smallest sample), and OTU abundances were calculated as the whole-number means across iterations. Differences in bacterial community were visualized by nonmetric dimensional scaling plots (nMDS, iters = 10 000) (Shepard, 1966) of Bray-Curtis (Bray and Curtis, 1957) and Jaccard (Jaccard, 1912) similarity (beta-diversity) indices, also calculated in mothur.

All other statistical analyses were carried out in R (Oksanen *et al.*, 2015), and data are expressed as the mean ± SE and considered significant if *P* < 0.05. Differences in taxonomic profiles were assessed at the phylum, order and OTU levels. Due to uneven sampling, analysis of similarity (ANOSIM) was used to compare community structure (Bray-Curtis) (Bray and Curtis, 1957) and community composition (Jaccard) (Jaccard, 1912) across all four stages. Samples were randomized with respect to weaning stage and tested to ensure true significance. Similarity percentages (SIMPER) analyses were then used to determine the contributions of taxonomic groups to differences observed in the ANOSIM. Permutational analysis of multivariate dispersions (PERMDISP) was used to test for heterogeneity of community structure and composition in weaning stages, and the general linearized model (ANOVA) was used to determine if alpha diversity and relative abundance of taxa differed with respect to weaning stage.

## Results

### The developing red panda microbiota has low diversity

For all samples, a total of 467 410 raw and 180 411 high-quality 16S rRNA sequences (12 024 ± 1844 per sample) were generated using Illumina MiSeq paired-end sequencing (see Table S1). A Good’s coverage value of >0.99 (see Table S1) and a leveling off of rarefaction curves (see Fig. S1) indicated that sequencing was adequate to detect the majority of bacterial diversity present in all samples. A 97% operational taxonomic unit (OTU) analysis corresponding to species-level classification (Schloss and Handelsman, 2005) identified 170 OTUs across all samples with values ranging from 11 to 77 OTUs per sample (see Table S1 and Supplementary Data S1).

**Figure 1:**
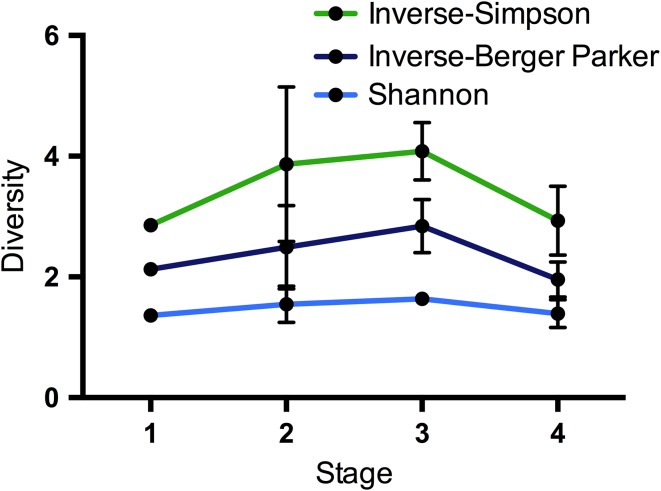
Mean ± SE Shannon, inverse-Simpson and inverse-Berger–Parker diversity indices across all weaning stages.

Each sample’s microbiota displayed low diversity and was dominated by a single OTU (Berger–Parker: 0.49 ± 0.041, see Table S1). Although not significant (ANOVA, *P* > 0.05), samples increased slightly in diversity (inverse-Berger–Parker, Shannon and inverse-Simpson indices) during weaning (Stage 2, *n* = 3), reaching the highest observed levels post-weaning for both inverse-Simpson and inverse-Berger–Parker (Stage 3, *n* = 3). Adult samples (Stage 4, *n* = 6) showed decreased diversity at levels similar to pre-weaning (Stage 1, *n* = 3) (Fig. 1, see Table S1).

### Red pandas have a core bacterial community maintained over time

**Figure 2:**
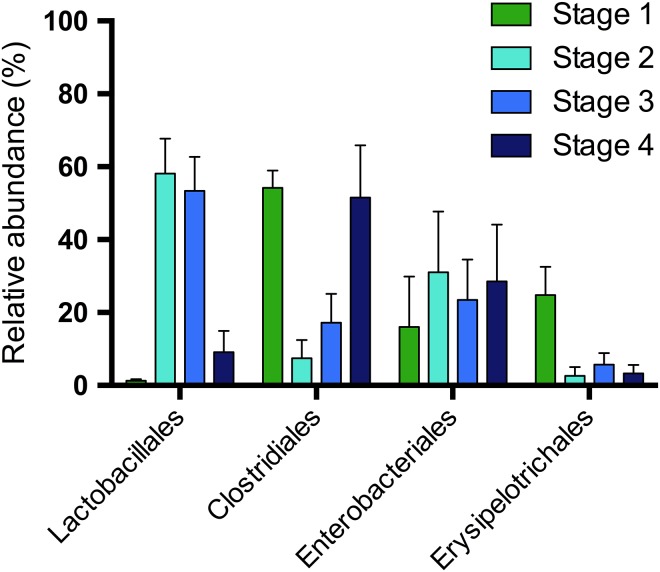
Mean + SE relative sequence abundance of taxonomic orders found at ≥1.0% relative sequence abundance for each weaning stage.

Analysis of the sequences from all samples revealed the presence of five phyla, with 71 ± 6.9% belonging to Firmicutes, 27 ± 7.1% to Proteobacteria, and <1.0% each to Actinobacteria, Bacteroidetes and Tenericutes. At lower taxonomic classifications, bacterial classes with >1.0% relative sequence abundance included Clostridia (36 ± 7.8%), Bacilli (27 ± 7.1%), Gammaproteobacteria (27 ± 7.2%) and Erysipelotrichia (7.9 ± 2.8). At the order level, those with >1.0% representation were Clostridiales (36 ± 7.8%), Lactobacillales (26 ± 7.2%), Enterobacteriales (26 ± 7.3%) and Erysipelotrichales (7.9 ± 2.8%, Fig. 2). At the family and genus level, 93% and 45% of sequences, respectively, were annotated.

A total of 170 OTUs were found in the dataset, with 43 OTUs found appreciably abundant (relative abundance ≥1.0%) and present in more than one weaning stage (see Supplementary Data S1 and Table S2). Nine of these OTUs were present in all four stages, and of these, five were highly abundant, including an *Escherichia-Shigella* (23 ± 7.1%, OTU001), a *Streptococcus* (13 ± 5.3%, OTU002), a *Clostridium* (11 ± 4.9%, OTU003), an unclassified Clostridiaceae (10 ± 4.3%, OTU004) and a *Turicibacter* (7.9 ± 2.9%, OTU005). The other four OTUs were less abundant and classified as a *Lactococcus* (4.8 ± 2.6%, OTU008), an *Enterococcus* (2.7 ± 0.82%, OTU010), a *Klebsiella* (1.4 ± 0.70%, OTU011) and a *Psychrobacter* (0.71 ± 1.0%, OTU015). In total, these nine shared OTUs accounted for 74 ± 6.6% of the sequences within each sample.

### The young red panda microbiota differs according to weaning stage

**Figure 3:**
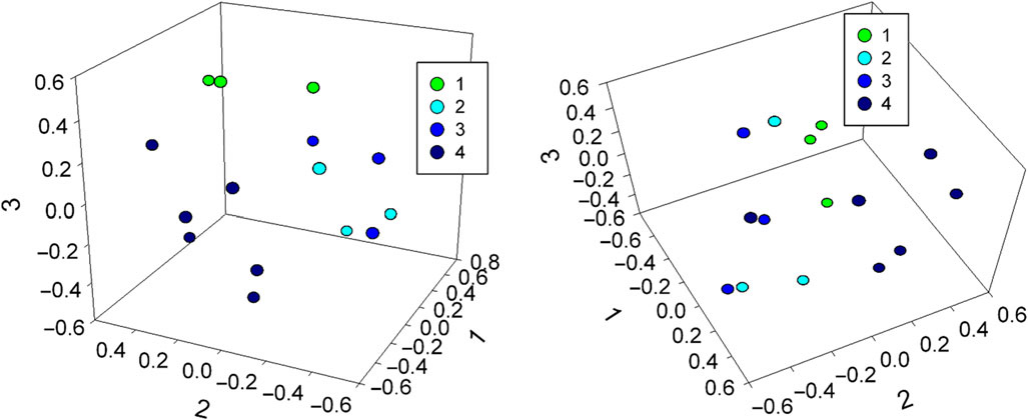
Three-dimensional nonmetric multi-dimensional scaling analysis of community, (**a**) composition (Jaccard, lowest stress: 0.20, R-square: 0.64) and (**b**) structure (Bray-Curtis, lowest stress: 0.11, R-square: 0.90) for weaning stages.

To determine if the GIT microbiota differed across weaning stages, we compared total bacterial community structure (Bray-Curtis) and composition (Jaccard). Changes in the overall bacterial community were visualized by nonmetric multi-dimensional scaling (nMDS) (Fig. 3). During weaning (Stages 2 and 3), samples clustered together, and therefore, were more similar to each other compared to either Stage 1 or 4, which had distinct clustering for both community composition (Fig. 3a) and structure (Fig. 3b).

Statistical analyses revealed that bacterial composition and structure did not vary at the phylum level (ANOSIM, *P* = 0.95 and *P* = 0.96, respectively). However, bacterial composition varied significantly at the order level (ANOSIM, *P* = 0.049) with two orders, Lactobacillales and Erysipelotrichales, found to change in relative abundances across weaning stages (ANOVA *P* = 0.00029 and *P* = 0.0090, respectively). Community composition and structure were also found to vary at the OTU level (ANOSIM, *P* = 0.0086 and *P* = 0.0077, respectively), with Stage 4 (*n* = 6) having higher inter-sample variation than Stage 1 (*n* = 3) as evidenced by more disperse points in the nMDS plots (Fig. 3; PERMDISP, *P* = 0.017 and *P* = 0.032, respectively). However, this increased dispersal may be due to the larger sample size of Stage 4 compared to Stages 1–3. Seventeen OTUs significantly contributed to the variation seen among weaning stages (SIMPER > 1.0%), and all but one were present in more than one weaning stage (see Table S2), indicating important changes in abundance as opposed to presence/absence. Randomized controls were not significantly different at any taxonomic level (see Table S3).

Within the orders and OTUs identified by SIMPER analysis, the Erysipelotrichales and a single OTU classified within this order as *Turicibacter* (OTU005) varied by weaning stage (ANOVA, *P* = 0.00029 and *P* = 0.0093, respectively). Both the order and OTU decreased significantly from Stage 1 (*n* = 3) to Stage 2 (*n* = 3) and then remained low in Stage 3 (*n* = 3) and Stage 4 (*n* = 6) with slightly higher abundances in Stage 3 (*n* = 3) (Fig. 2, see Table S4). The order Lactobacillales (*P* = 0.00029), but no OTUs within this order, varied by stage (ANOVA, *P* > 0.05), though a number of Lactobacillales OTUs were identified by SIMPER analysis to contribute to variation (see Table S4). The relative abundance of the Lactobacillales order increased from Stage 1 to 2 then slightly decreased by Stage 3 (all stages, *n* = 3) and dramatically decreased further by Stage 4 (*n* = 6) (Fig. 2). OTUs within the order that followed the same trend were an *Enterococcus* (OTU010), a *Lactobacillus* (OTU017), a *Lactococcus* (OTU014), a *Streptococcus* (OTU002) and a *Weissella* (OTU021) (see Table S4). Divergent Lactobacillales OTUs were a *Lactococcus* (OTU008) and *Leuconostoc* (OTU009) that instead, continued to increase from Stage 2 (*n* = 3) to Stage 3 (*n* = 3) before decreasing by Stage 4 (*n* = 6) (see Table S4).

Although not significant (ANOVA, *P* > 0.05), Clostridiales appeared to display an inverse relationship to Lactobacillales, with higher levels observed in Stage 1 (*n* = 3) and Stage 4 (*n* = 6) (Fig. 2). However, no OTUs within this order displayed the same trend. Instead, trends among SIMPER-identified OTUs significantly varied across the Clostridiales order with a *Clostridium* (OTU003) and an unclassified Peptostreptococcaceae (OTU012) initially decreased from Stage 1 (*n* = 3) to Stage 2 (*n* = 3) then increased in Stage 3 (*n* = 3) before decreasing by Stage 4 (*n* = 6) to levels similar to Stage 2 (*P* < 0.001 and *P* = 0.0081, respectively). A *Sarcina* (OTU007) and an unclassified Clostridiaceae (OTU006) were at or near zero until dramatically increasing in Stage 4 (*n* = 6) while a different unclassified Clostridiaceae (OTU004) continually increased across the four stages. Finally, an unclassified Lachnospiraceae (OTU018) initially increased from Stage 1 (*n* = 3) to Stage 2 (*n* = 3) and then decreased across Stage 3 (*n* = 3) and Stage 4 (*n* = 6) (see Table S4).

Several OTUs within Enterobacteriales contributed to the observed variation between weaning stages (SIMPER ≥ 1.0%) even though this order showed minimal variation overall (Fig. 2). Both a *Klebsiella* (OTU011) and an unclassified Enterobacteriaceae (OTU013) increased from Stage 1 (*n* = 3) to Stage 2 (*n* = 3) and then decreased across Stage 3 (*n* = 3) and Stage 4 (*n* = 6) to be at or near zero abundance. In contrast, an *Escherichia-Shigella* (OTU001) did not continue to decrease in the later stages but instead, increased to its highest levels in Stage 4 (*n* = 6) (see Table S4).

## Discussion

The establishment of an adult gut microbiota is vital for the survival of herbivores as they rely on these communities for nutrient extraction from their exclusively plant-based diets (Bergman, 1990). Weaning, and the resulting anatomical changes that occur in developing herbivores, is thought to drive the establishment of these gut communities and enable the host’s survival as an adult (Koenig *et al.*, 2011; Edrington *et al.*, 2012; Jami *et al.*, 2013; Rey *et al.*, 2013). Red pandas are an exception, as they are herbivorous carnivores that lack specialized GIT adaptations (Davis, 1964). Thus, they likely develop their gut microbiota differently than other herbivores. Challenges related to nutritional inadequacies, such as insufficient maternal care and bamboo quality, may contribute to the high cub mortality of red pandas in captivity (Loeffler, 2011), and thus understanding how the GIT microbiota is established may provide insights into this problem. Here, we conducted an initial characterization of the GIT bacterial microbiota of two captive red pandas across weaning and into adulthood using high-throughput 16S rRNA Illumina sequencing.

Overall, we found that the young red panda GIT had low diversity (see Table S1) and was dominated by bacteria in the phylum Firmicutes with lesser contributions from the Proteobacteria. This composition is consistent with previous reports of captive adult red pandas, and to some extent, wild adult red pandas, which also have a significant presence of Bacteroidetes (Kong *et al.*, 2014). Across weaning, the GIT microbiota of red panda cubs (Stages 1–3, all *n* = 3) had an abundant core community defined by 9 OTUs that comprised 60–97% of individual samples. Therefore, it appears that the red panda harbours a large proportion of the microbiota necessary for survival on a plant-based diet (Stages 3 and 4, *n* = 3 and 6, respectively) as early as 1 month of age (Stage 1, *n* = 3). We posit that initial colonization by these organisms may occur during birth, or very early on in life through maternal contact, similar to humans (Mackie *et al.*, 1999; Vaishampayan and Kuehl, 2010; Vallès *et al.*, 2014), and failure to acquire early communities may contribute to cub mortality in captivity.

The abundances of OTUs within the core microbiota varied significantly between stages and individual samples, particularly in adults (Stage 4, *n* = 6), where the core comprised anywhere from 13% to 93% of the reads per samples. This suggests that, while cubs possess taxa similar to adults, there are important selective factors that contribute to the development of adult microbial abundances. In cubs, these factors likely include dietary changes during weaning, as demonstrated by the increased apparent gut microbial diversity in red pandas that follows the shift to a more diverse diet of milk and bamboo. In adults (Stage 4, *n* = 6), maintenance of a strictly bamboo diet appears to cause further selection, as shown by the decrease in diversity from Stage 3 to 4 in this study (*n* = 3 and 6, respectively, Fig. 1). Developmental changes that occur post-weaning may have caused divergence between the male and female samples, leading to the different core communities observed. As such, developmental changes were not considered in this analysis, and our understanding of the divergence from Stage 3 to 4 is limited to observed differences in gut microbiota with respect to dietary changes (*n* = 3 and 6, respectively).

While the same core OTUs dominated the red panda gut microbiota in most samples, the overall community structure and composition changed across weaning with fluctuations in abundances across different weaning stages (Fig. 3, see Table S4). For example, we found that one member of the core community, a *Turicibacter* OTU (order Erysipelotrichales), is highly abundant, indicating that it may be important in very young cubs (Arumugam *et al.*, 2011), while several distinct OTUs within the order Clostridiales appear to persist as the animals age. *Turicibacter* may play an important role in early development of the host immune system as this highly heritable taxon has been linked to host immunity and found to have host-genetic associations in both humans and mice (Kellermayer *et al.*, 2011; Dimitriu *et al.*, 2013; Goodrich *et al.*, 2016). In contrast, OTUs within the Clostridiales were associated to changes across all weaning stages. The Clostridiales, which have a wide range of metabolic abilities, including species that are proteolytic, saccharolytic and cellulolytic (Wiegel *et al.*, 2006), have been reported previously in other herbivores (Fonty *et al.*, 1987; Rey *et al.*, 2013). We speculate that changes in the abundance of different members of the Clostridiales in developing red pandas are due to available dietary substrates, with Stages 2 and 3 (both, *n* = 3) serving as a transition between the milk-degrading proteolytic and saccharolytic species (Stage 1, *n* = 3) and the bamboo-degrading cellulolytic species (Stage 4, *n* = 6). In particular, the Clostridiales member *Sarcina* may be an important fibrolytic organism in the red panda GIT as it was only found in adult animals and is also associated with humans consuming a vegetarian diet (Crowther, 1971).

During the weaning transition, OTUs within the orders Lactobacillales and Enterobacteriales were highly abundant in Stages 2 and 3 (both, *n* = 3), but at low to not detectable abundances in Stages 1 and 4 (*n* = 3 and 6, respectively; see Table S4). These low adult abundances are in agreement with previous work in other captive red pandas (Kong *et al.*, 2014). Lactobacillales (Reid and Burton, 2002; Salvetti *et al.*, 2013) and Enterobacteriales (Ghanbarpour and Kiani, 2013; Ebrahimi *et al.*, 2014) are common members of the mammalian GIT and are known to ferment a variety of milk and lignocellulosic breakdown products into short-chain fatty acids (Reid and Burton, 2002), which serve as an energy source for the host (Makarova *et al.*, 2006). As these taxa are only highly abundant in the red panda during weaning, they may be opportunistic and take advantage of new niches appearing with the introduction of new dietary components, but fail to persist long-term on the more difficult to degrade adult bamboo diet. For example, members of the Lactobacillales help to maintain active immunity in ruminants following the loss of passive immunity acquired from colostrum (Malmuthuge *et al.*, 2013). Several Lactobacillales OTUs increased in abundance as the cubs transitioned from Stage 1 to 2 (both, *n* = 3), which coincided with a dietary shift to more fibrous substrates, and the roles this order plays in both substrate fermentation and immune function maintenance likely contributes to the importance of these species in the GIT. During post-weaning (Stage 3, *n* = 3), the Lactobacillales OTUs fluctuated, with some increasing in abundance while others decreased. Those OTUs that decreased, which include an *Enterococcus*, a *Lactococcus*, a *Lactobacillus*, a *Streptococcus* and a *Weissella*, are known to have slightly different substrate preferences, including those derived from milk, whereas OTUs that increased, like *Leuconostoc*, are known to utilize lignocellulosic breakdown products (Ohara, Owaki and Sonomoto, 2006).

Overall, our study demonstrates that the GIT bacterial community of two developing red pandas is significantly different with respect to weaning stage, with few taxonomic groups responsible for these differences. Specifically, taxa in the core GIT microbiota are established early on, but changes in diet during weaning, and possibly physiological changes post-weaning, further shape the GIT community by driving the relative abundances of these taxa. Our work reveals that a succession of microbes occurs across weaning stages, and we propose that this is due to the shift in diet that occurs as these red pandas transition from a milk- to a plant-based diet. Due to the observed early establishment of the core microbiota in the two healthy red pandas used in our study, it appears proper development and cub survival may rely on the initial colonization of a core microbial community. We acknowledge that this exploratory study contains a small sample size, and we are unable to draw any strong conclusions as to what factors shape the gut microbiota of young red pandas. Given that there are few opportunities to study red panda cubs of the same age in the same geographical location, the information presented here is valuable as an initial study. With such high levels of cub mortality in captive red pandas, further work on a larger group of young red pandas, particularly those suffering nutritional issues, is needed to more fully understand the role of the GIT microbiota in host health and development.

## Supplementary Material

Supplementary Data 1

Supplementary Figure 1

Supplementary Table 1

Supplementary Table 2

Supplementary Table 3

Supplementary Table 4

## Data Accessibility

All 16S rRNA gene sequencing information was deposited into the National Center for Biotechnological Information’s Short Read Archive under Accession Number SRP077938.
