# Supplementary figures and images for: Dietary changes during weaning shape the gut microbiota of red pandas (Ailurus fulgens)
Source: Conserv Physiol. 2018 Jan 6;6(1):cox075. doi: 10.1093/conphys/cox075 (PMC5772406; doi:10.1093/conphys/cox075)

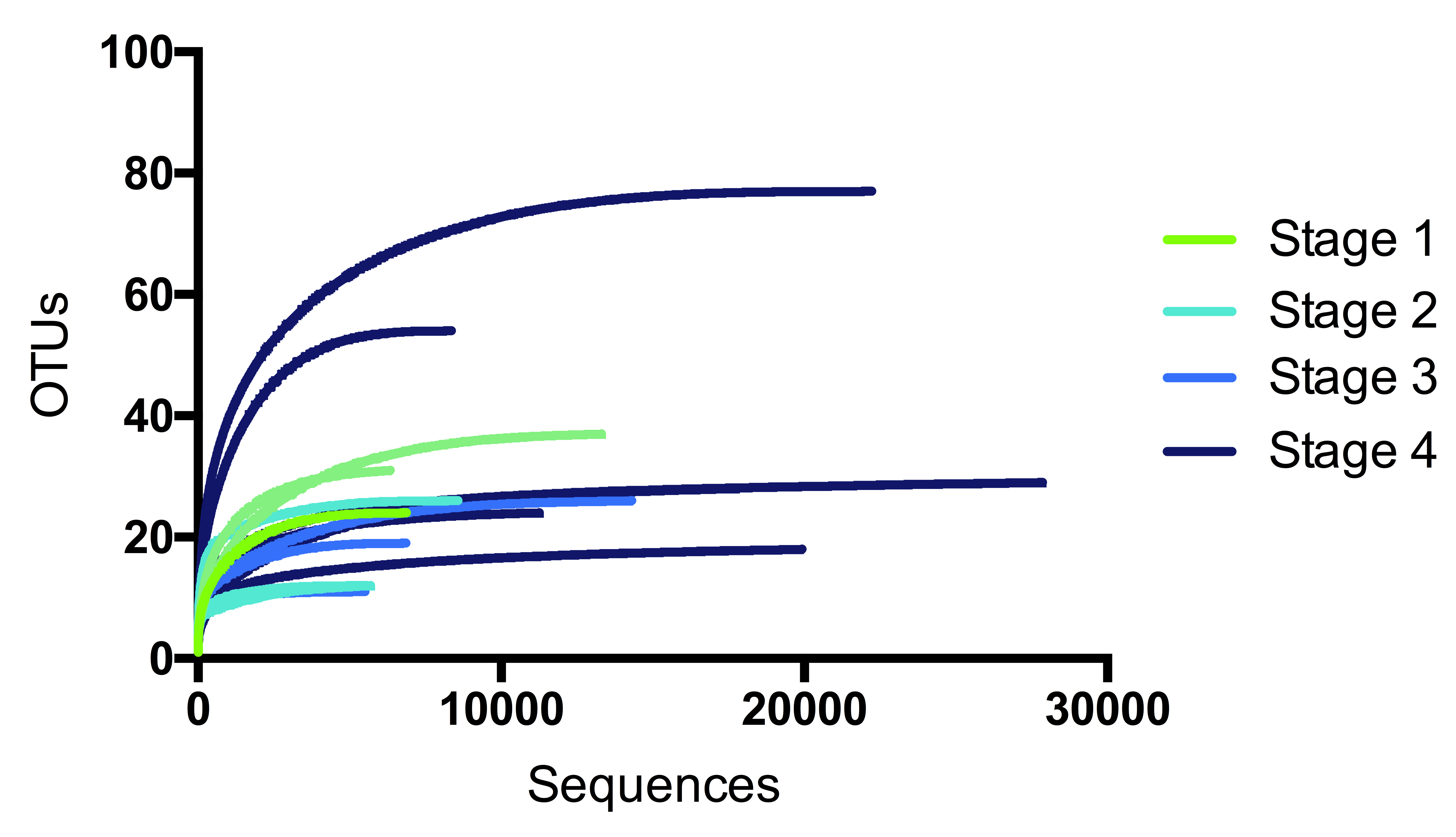

Supplement: Supplementary Figure 1 [file cox075williamssf1.png]
