# Supplementary material for: Dietary changes during weaning shape the gut microbiota of red pandas (Ailurus fulgens)
Source: Conserv Physiol. 2018 Jan 6;6(1):cox075. doi: 10.1093/conphys/cox075 (PMC5772406; doi:10.1093/conphys/cox075)
Supplement: Supplementary Table 1 [file cox075williamssuppts1.doc]

**Table S1.** Number of sequences, estimated coverage, diversity and OTU richness in each sample.

Mean values with standard error given.

| **Sample** | **16S rRNA reads** | | **Community Richness** | | | | **Community Diversity** |
| --- | --- | --- | --- | --- | --- | --- | --- |
|  | **Total Reads** | **High-quality Reads** | **Inverse**  **Berger-Parker** | **Shannon** | **Inverse-Simpson** | **Unique OTUs** | **Good’s Coverage** |
| Total (n=15) | 31161 ± 3018 | 12027 ± 1816 | 2.3 ± 0.19 | 1.5 ± 0.010 | 3.3 ± 0.33 | 126 | > 0.99 |
| Stage 1 (n=3) | 38059 ± 6962 | 8840 ± 2242 | 2.1 ± 0.085 | 1.4 ± 0.022 | 2.9 ± 0.034 | 29 | > 0.99 |
| Stage 2 (n=3) | 25809 ±4187 | 6616 ± 978 | 2.5 ± 0.69 | 1.5 ± 0.30 | 3.9 ± 1.3 | 6 | > 0.99 |
| Stage 3 (n=3) | 32801 ± 10221 | 8892 ± 2733 | 2.8 ± 0.44 | 1.6 ± 0.062 | 4.1 ± 0.48 | 10 | > 0.99 |
| Stage 4 (n=6) | 29567 ± 6522 | 17894 ± 2927 | 2.0 ± 0.31 | 1.4 ± 0.31 | 2.9 ± 0.73 | 81 | > 0.99 |
