# Supplementary material for: Dietary changes during weaning shape the gut microbiota of red pandas (Ailurus fulgens)
Source: Conserv Physiol. 2018 Jan 6;6(1):cox075. doi: 10.1093/conphys/cox075 (PMC5772406; doi:10.1093/conphys/cox075)
Supplement: Supplementary Table 2 [file cox075williamssuppts2.doc]

**Table S2.** Abundant OTUs significant to red panda gut microbiota.

| **OTU** | **Phylum** | **Lowest taxonomic classification** | **Stage 1** | **Stage 2** | **Stage 3** | **Stage 4** |
| --- | --- | --- | --- | --- | --- | --- |
| 010* | Firmicutes | *Enterococcus* | ☐ | ◼ | ◼ | ◼ |
| 008* | Firmicutes | *Lactococcus* | ☐ | ☐ | ☐ | ◼ |
| 002* | Firmicutes | *Streptococcus* | ◼ | ◼ | ◼ | ☐ |
| 003*^†^ | Firmicutes | *Clostridium* | ◼ | ☐ | ◼ | ◼ |
| 004* | Firmicutes | Clostridiaceae | ☐ | ☐ | ☐ | ◼ |
| 005*^†^ | Firmicutes | *Turicibacter* | ◼ | ☐ | ☐ | ◼ |
| 001* | Proteobacteria | *Escherichia-Shigella* | ◼ | ◼ | ◼ | ◼ |
| 011* | Proteobacteria | *Klebsiella* | ☐ | ◼ | ☐ | ☐ |
| 015 | Proteobacteria | *Psychrobacter* | ☐ | ☐ | ☐ | ◼ |
| 009* | Firmicutes | *Leuconostoc* | ☐ | ☐ | ☐ |  |
| 041 | Actinobacteria | *Arthrobacter* | ☐ | ☐ |  | ☐ |
| 022 | Firmicutes | *Jeotgalicoccus* | ☐ | ☐ |  | ☐ |
| 034 | Firmicutes | *Nosocomiicoccus* | ☐ | ☐ |  | ☐ |
| 027 | Firmicutes | *Eremococcus* | ☐ | ☐ |  | ☐ |
| 053 | Firmicutes | *Atopostipes* | ☐ | ☐ |  | ☐ |
| 062 | Firmicutes | *Clostridium* | ◼ |  | ☐ | ☐ |
| 012*^†^ | Firmicutes | Peptostreptococcaceae | ☐ |  | ☐ | ☐ |
| 066 | Firmicutes | Peptostreptococcaceae | ☐ |  | ☐ | ☐ |
| 054 | Actinobacteria | *Kocuria* |  | ☐ | ☐ | ☐ |
| 021* | Firmicutes | *Weissella* |  | ☐ | ☐ | ☐ |
| 014* | Firmicutes | *Lactococcus* |  | ☐ | ☐ | ☐ |
| 018* | Firmicutes | Lachnospiraceae |  | ☐ | ☐ | ☐ |
| 044 | Proteobacteria | Gammaproteobacteria |  | ☐ | ☐ | ☐ |
| 081 | Firmicutes | *Clostridium* | ☐ |  | ☐ |  |
| 025 | Firmicutes | *Atopostipes* | ◼ |  |  | ☐ |
| 020 | Firmicutes | *Staphylococcus* | ☐ |  |  | ☐ |
| 082 | Firmicutes | *Trichococcus* | ☐ |  |  | ☐ |
| 051 | Firmicutes | *Streptococcus* | ☐ |  |  | ☐ |
| 078 | Proteobacteria | Enterobacteriaceae | ☐ |  |  | ☐ |
| 023 | Firmicutes | Clostridiaceae | ◼ |  |  | ☐ |
| 006* | Firmicutes | Clostridiaceae | ☐ |  |  | ◼ |
| 024 | Firmicutes | Peptostreptococcaceae | ◼ |  |  | ☐ |
| 017* | Firmicutes | *Lactobacillus* |  | ☐ | ☐ |  |
| 043 | Firmicutes | *Lactobacillus* |  | ☐ | ☐ |  |
| 032 | Firmicutes | *Pediococcus* |  | ☐ | ☐ |  |
| 064 | Firmicutes | Ruminococcaceae |  | ☐ | ☐ |  |
| 050 | Firmicutes | Erysipelotrichaceae |  | ☐ | ☐ |  |
| 013* | Proteobacteria | Enterobacteriaceae |  | ☐ | ☐ |  |
| 029 | Firmicutes | *Aerococcus* |  | ☐ |  | ☐ |
| 093 | Proteobacteria | *Acinetobacter* |  | ☐ |  | ☐ |
| 046 | Firmicutes | *Carnobacterium* |  | ☐ |  | ☐ |
| 031 | Firmicutes | *Staphylococcus* |  |  | ☐ | ☐ |
| 106 | Proteobacteria | *Acinetobacter* |  |  | ☐ | ☐ |
| 007* | Firmicutes | *Sarcina* |  |  |  | ◼ |

◼ denotes that all samples within a stage contained the specified OTU

☐denotes ≤ 2 samples within each stage contained the specified OTU.

*Contributes to variation (SIMPER)

^†^Significant (ANOVA)
