# Supplementary material for: Dietary changes during weaning shape the gut microbiota of red pandas (Ailurus fulgens)
Source: Conserv Physiol. 2018 Jan 6;6(1):cox075. doi: 10.1093/conphys/cox075 (PMC5772406; doi:10.1093/conphys/cox075)
Supplement: Supplementary Table 3 [file cox075williamssuppts3.doc]

**Table S3**. P-values of weaning stage and randomized control differences with respect to community structure and composition at the phyla, family, and OTU level.

|  | **Phyla** | | **Family** | | **OTU** | |
| --- | --- | --- | --- | --- | --- | --- |
|  | Stage | Control | Stage | Control | Stage | Control |
| **Bray-Curtis** | 0.96 | 0.63 | 0.049* | 0.57 | 0.0086* | 0.27 |
| **Jaccard** | 0.95 | 0.64 | 0.046* | 0.56 | 0.0077* | 0.27 |

*Significant
