# Supplementary material for: Dietary changes during weaning shape the gut microbiota of red pandas (Ailurus fulgens)
Source: Conserv Physiol. 2018 Jan 6;6(1):cox075. doi: 10.1093/conphys/cox075 (PMC5772406; doi:10.1093/conphys/cox075)
Supplement: Supplementary Table 4 [file cox075williamssuppst4.doc]

**Table S4.** Average percent relative abundances of OTUs that differ by stage.

| **OTU** | **Phylum** | **Lowest taxonomic classification** | **Stage 1** | **Stage 2** | **Stage 3** | **Stage 4** |
| --- | --- | --- | --- | --- | --- | --- |
| 010* | Firmicutes | *Enterococcus* | 0.10 ± 0.08 | 5.8 ± 2.3 | 3.0 ± 1.2 | 2.2 ± 1.3 |
| 008* | Firmicutes | *Lactococcus* | 0.10 ± 0.09 | 8.6 ± 8.6 | 14 ± 9.2 | 0.44 ± 0.30 |
| 002* | Firmicutes | *Streptococcus* | 0.39 ± 0.30 | 29 ± 17 | 25 ± 15 | 5.7 ± 5.5 |
| 003*^†^ | Firmicutes | *Clostridium* | 45 ± 3.7^a^ | 0.40 ± 0.40^b^ | 7.4 ± 7.4^b^ | 0.52 ± 0.24^b^ |
| 004* | Firmicutes | Clostridiaceae | 0.04 ± 0.04 | 5.8 ± 5.8 | 8.1 ± 5.9 | 19 ± 9.4 |
| 005*^†^ | Firmicutes | *Turicibacter* | 25 ± 7.7^a^ | 2.6 ± 2.5^b^ | 5.6 ± 3.3^b^ | 3.3 ± 2.4^b^ |
| 001* | Proteobacteria | *Escherichia-Shigella* | 16 ± 14 | 26 ± 15 | 15 ± 8.2 | 28 ± 16 |
| 011* | Proteobacteria | *Klebsiella* | 0.04 ± 0.04 | 4.1 ± 1.7 | 3.0 ± 2.6 | 0.03 ± 0.02 |
| 009* | Firmicutes | *Leuconostoc* | 0.01 ± 0.01 | 4.9 ± 4.9 | 9.3 ± 9.3 | ND |
| 012*^†^ | Firmicutes | Peptostreptococcaceae | 6.2 ± 2.7^a^ | ND^b^ | 0.80 ± 0.80^b^ | 0.03 ± 0.02^b^ |
| 021* | Firmicutes | *Weissella* | ND | 1.3 ± 1.3 | 0.36 ± 0.36 | 0.05 ± 0.03 |
| 014* | Firmicutes | *Lactococcus* | ND | 5.3 ± 5.3 | 0.48 ± 0.48 | 0.01 ± 0.01 |
| 018* | Firmicutes | Lachnospiraceae | ND | 1.3 ± 1.3 | 0.68 ± 0.68 | 0.10 ± 0.10 |
| 006* | Firmicutes | Clostridiaceae | 0.01 ± 0.01 | ND | ND | 19 ± 8.8 |
| 017* | Firmicutes | *Lactobacillus* | ND | 1.7 ± 1.0 | 0.68 ± 0.68 | ND |
| 013* | Proteobacteria | Enterobacteriaceae | ND | 1.2 ± 0.71 | 5.8 ± 5.8 | ND |
| 007* | Firmicutes | *Sarcina* | ND | ND | ND | 13 ± 4.8 |

Mean ± SEM. ND: not detected

*Contributes to variation (SIMPER)

^†^Significant (ANOVA). Values in the same row with different letters are significantly different (P < 0.05).
